# Risk Factors and Prognostic Impact of Postoperative Complications in Patients with Advanced Gastric Cancer Receiving Neoadjuvant Chemotherapy

**DOI:** 10.3390/curroncol29090511

**Published:** 2022-09-10

**Authors:** Hong Yu, Li Xu, Songcheng Yin, Jianlong Jiang, Chunhong Hong, Yulong He, Changhua Zhang

**Affiliations:** 1 Digestive Disease Center, The Seventh Affiliated Hospital of Sun Yat-Sen University, Shenzhen 518107, China; 2 Emergency and Disaster Medicine Center, The Seventh Affiliated Hospital of Sun Yat-Sen University, Shenzhen 518107, China

**Keywords:** neoadjuvant chemotherapy, postoperative complication, gastric cancer

## Abstract

Background: Neoadjuvant chemotherapy is important to improve the prognosis of patients with advanced gastric cancer. However, it may result in postoperative complications (POCs). The aim of this study is to evaluate risk factors and prognostic impact of POCs in patients receiving neoadjuvant chemotherapy. Methods: We retrospectively collected clinical information of patients who underwent curative gastrectomy after receiving neoadjuvant chemotherapy between 2011 and 2018. Overall survival (OS) was analyzed using the Kaplan–Meier method. Logistic regression and Fisher’s exact test were used to evaluate risk factors for complications. Results: A total of 176 patients were included in our study. The 3-year OS rates for the complication group (*n* = 30) and non-complication group (*n* = 146) were 36.7% and 52.7%, respectively (*p* = 0.0294). Age, BMI, multivisceral resection and operation time were independent risk factors for POCs in patients. Patients with multivisceral resection were more likely to suffer from grade III-IV complications (*p* = 0.026). Inflammation complications might occur in patients with high BMI (*p* = 0.017). Low preoperative albumin seemed to be a risk factor for leakage complications (*p* = 0.033). Conclusions: Our study revealed that patients with POCs had a poor prognosis and we identified the risk factors for complications so that POCs can be avoided in time.

## 1. Introduction

Gastric cancer (GC) is the fifth most commonly diagnosed malignancy and the fourth cause of cancer-related mortality globally [1]. Despite advances in technology and increasing attention on screening for early cancer, the prognosis of patients with advanced GC remains poor. Radical gastrectomy, accompanied by D2 or even D3 lymph node dissection, is currently the most effective treatment for GC. However, 20–40% of patients will inevitably develop postoperative recurrences or metastases [2]. While patients are undergoing surgery, surgical team sometimes observe small metastases scattered in the abdominal cavity or even full-blown invasion of surrounding organs that were not foreseeable using modern imaging technologies, indicating a poor prognosis. Neoadjuvant chemotherapy (NAC) is important in improving the prognosis of patients with advanced GC and has been recommended in the guidelines ever since its acceptance [3,4]. NAC can increase the possibility of curative resection by downstaging the tumor, eliminating early micrometastases, and allowing an in vivo response assessment of treatment [5]. In the past decade, new therapeutic targets for advanced GC have emerged, especially HER2, which have saved countless desperate patients [6]. Trastuzumab and its derivatives have been widely used in clinical practice, and many clinical trials are exploring the possibility of expanded indications. On the other hand, understanding the biology of the tumor microenvironment is important, as well as the interactions between tumor cells and surrounding immune cells, as they can have an impact on tumor therapy [7]. Tumor-associated macrophages have attracted much attention because of their important role in predicting prognosis and drug resistance. The consideration of microsatellite instability (MSI) in diagnosis is a relatively recent development in the examination of patients with potentially resectable disease. These advancements drive updates in current systemic therapy approaches and allow for the identification of populations most likely to benefit from immunotherapy [8].

In spite of the apparent survival advantage of NAC, it may result in minor or major postoperative complications (POCs) due to chemotherapeutic damage to gastric tissue or reduced surgical tolerances of patients [9]. NAC can cause bone marrow suppression and postoperative malnutrition, leading to decreased immune function and increased surgical complications. Several studies have shown that POC can have a negative impact on the prognosis of patients, especially in patients with severe infectious complications [10,11,12]. Therefore, it is particularly paramount to grasp the indication of NAC accurately and reduce POCs as much as possible.

The aim of this study is to evaluate effect of POCs on patients’ prognosis and analyze risk factors for NAC complications. We hope that this study will provide guidance for clinical decisions to improve the prognosis of patients with advanced GC receiving neoadjuvant therapy.

## 2. Materials and Methods

### 2.1. Data Collection

We retrospectively collected clinical information of patients who underwent curative (R0) gastrectomy after receiving NAC between 2011 and 2018 at the First Affiliated Hospital of Sun Yat-sen University (FAHSYSU) in Guangzhou, China. The following inclusion criteria were enforced: (1) patient diagnosed with advanced GC and treated for the first time; (2) gastric adenocarcinoma confirmed via postoperative pathology; (3) preoperative imaging examination showed no distant metastasis; and (4) complete clinical information available. The exclusion criteria were as follows: (1) history of any other tumor; (2) remnant GC; and (3) history of abdominal surgery. Characteristics, tumor pathological factors, treatment information, and surgical records of the patients were obtained from the FAHSYSU database, including gender, age, body mass index (BMI), extent of gastrectomy, TNM stage, multivisceral resection, operation time, preoperative laboratory data (hemoglobin, neutrophil, platelet, and albumin), and severity of POCs. We reevaluated the patients according to the eighth American Joint Committee on Cancer TNM staging system. Our study was in compliance with the Declaration of Helsinki (as revised in 2013). This study was approved by the Medical Ethics Committee of the Seventh Affiliated Hospital of Sun Yat-sen University (No.KY-2020-024-01). Individual consent for this retrospective analysis was waived.

### 2.2. Evaluation of POCs

Any complications occurring within one month after surgery were recorded, including intra-abdominal bleeding or infections, ileus, anastomotic leakage, or stenosis and pulmonary infection. The severity of POCs was assessed in accordance with the Clavien–Dindo classification system [13]. If a patient had more than two concurrent complications, the higher-grade complication was chosen. We divided patients with NAC into two groups: those with complications (C group) and those without complications (NC group).

### 2.3. Follow-Up and Study End Points

After curative gastrectomy, all patients would be evaluated every 3 months in the first 2 years and every 6 months in the next 3 years. Follow-up was done through phone calls or outpatient visits to update on relapse or death. The last follow-up date was December 2021.

Overall survival (OS) was the study end point, which was defined as the duration from surgery to the date of death or last follow-up.

### 2.4. Statistical Analysis

OS rates and survival curves were evaluated according to the Kaplan–Meier method and were compared using the log-rank test. *p* values < 0.05 were considered statistically significant. Factors that predicted POCs were estimated using binary logistic regression analysis. Variables with *p* < 0.1 were further allowed to enter into the multivariate analyses. Categorical variables were analyzed by using the chi-square and Fisher’s exact tests. All statistical tests were conducted using SPSS version 24.0 (Chicago, IL, USA).

## 3. Results

### 3.1. Patient Characteristics

A total of 176 patients met the inclusion and exclusion criteria (Table 1). Of these patients, 112 (63.6%) were men and 133 (75.6%) were less than 65 years old. Most of these patients (86.9%) had a BMI of less than 25. Due to the late TNM and T stage of most patients, 69.9% of them underwent total gastrectomy. Some of them (26.7%) had multivisceral resection, and as a result, the operation lasted more than five hours in most cases (58.0%). Six patients had postoperative pathology of ypT0N0M0, indicating a pathological complete response. Unfortunately, micrometastases in the abdominal cavity were found during surgery in 32 patients due to poor response to chemotherapy, and the tumor progressed to stage IV. We still performed gastrectomy with resection of the single metastasis, believing that it would be beneficial for patients. In terms of preoperative laboratory data, most patients (58.0%) suffered from anemia due to GC or NAC. Among these patients, the overall POC rate was 17.0% (30/176).

**Figure 1:**
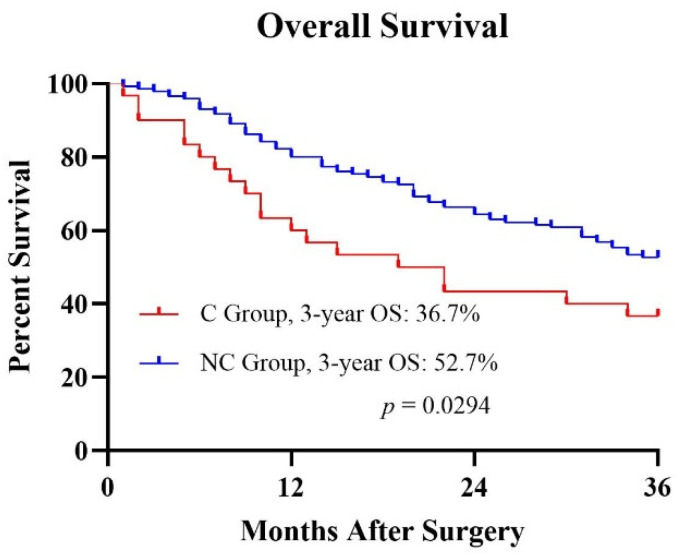
Kaplan–Meier curves of the OS of the two groups.

All follow-ups were more than 36 months after surgery. The 3-year OS rates for the C group (*n* = 30) and NC group (*n* = 146) were 36.7% and 52.7%, respectively (Figure 1). Kaplan–Meier survival analysis revealed that the POC was correlated with a poor prognosis (*p* = 0.0294).

### 3.2. Analysis of Risk Factors for POCs

With regard to risk factors for POCs, we used binary logistic regression analysis (Table 2). Univariate analysis showed that age, BMI, extent of gastrectomy, ypStage, ypT category, multivisceral resection, operation time, and preoperative albumin were risk factors for POCs in patients (*p* < 0.1). These factors with statistical significance in univariate analysis were included in multivariate analysis for further analysis. The results showed that age (*p* = 0.003), BMI (*p* = 0.005), multivisceral resection (*p* = 0.013), and operation time (*p* = 0.018) were independent risk factors for POCs in patients.

### 3.3. Risk Factors for Different Categories of POCs

Among the 30 complications, there were anastomotic leakages, pulmonary or abdominal infection, bleeding, bacteremia, and so on. We classified the POCs into different categories based on the Clavien–Dindo classification system, inflammation and leakage. According to the Clavien–Dindo classification system, 14 complications were grade I-II and 16 were grade III-IV (Table 3). As expected, patients with multivisceral resection were more likely to suffer from grade III-IV complications (*p* = 0.026). In terms of inflammation complications, there were 16 inflammation complications and 14 non-inflammation complications (Table 4). Inflammation complications may occur in patients with high BMI (*p* = 0.017). As far as leakage complications, 13 patients had leakage complications, either mild and requiring conservative treatment, or severe, which required surgery treatment (Table 5). Low preoperative albumin seemed to be a risk factor for leakage complications (*p* = 0.033).

## 4. Discussion

NAC has been included in guidelines for the treatment of advanced GC, expecting that it can downstage the tumor, reduce surgical difficulty, and improve prognosis of patients. However, due to the immense benefits of NAC, the adverse effects of NAC are not taken seriously. Our study demonstrated that patients receiving NAC had a poor prognosis once postoperative complications occurred. Moreover, it can be further inferred that patients with old age, high BMI, multivisceral resection, or long operation time were more likely to have POCs. Additionally, we have identified risk factors for different types of complications.

The MAGIC trial is the most recognized landmark study of perioperative chemotherapy, showing significant improvement in progression-free survival and OS in patients with advanced GC [14]. The FLOT4-AIO phase 2/3 multi-center clinical trial in Germany confirmed a higher proportion of patients with pathologically complete response and better OS in FLOT compared to ECF/ECX [9,15]. With the development of neoadjuvant therapy and the support of clinical research evidence, the safety of NAC has garnered more and more attention. Kang reported that incidence rates of surgery-related complications in a NAC group and surgery followed by adjuvant chemotherapy were 6% and 10% (*p* = 0.175), respectively [16]. The study by Liu suggested that the postoperative complications decreased significantly in laparoscopic gastrectomy following NAC compared to upfront laparoscopic gastrectomy [17]. Therefore, the safety of NAC has been fully guaranteed and it has been widely used in clinical practice.

To avoid POCs caused by neoadjuvant therapy, caregivers should pay greater attention during clinical treatment, because it may affect the recovery and prognosis of the patient. In the present study, patients with POCs after neoadjuvant therapy had a significantly worse prognosis than those without POCs (36.7% vs. 52.7%, *p* = 0.0294). Li revealed that the 3-year OS rates of patients experiencing major, minor, and no POC were 33.3%, 56.9%, and 62.1% (*p* = 0.023), respectively, probably due to the inability of patients with POCs to complete all intended multimodality therapy [18]. Over the past decades, scientists have been exploring the relationship between inflammation and tumor metastasis. In a murine model of liver metastasis, abdominal infection promoted liver metastasis, which may be related to the decrease in NK cell number and activity [19]. Toll-like receptors (TLRs) are sentinel receptors of the innate immune system that are activated during infection. Numerous types of solid cancer cells also express their own TLRs, which are also activated to improve their ability to metastasize [20]. Thus, avoiding POCs can improve the prognosis of patients with cancer.

Furthermore, we focused on the risk factors for POCs. Age and BMI are important factors controlling the occurrence of complications, as our study showed. This finding is similar to those of previous studies [21,22], confirming the reliability and authenticity of our study. In elderly patients, bone marrow suppression, decreased immune function, and malnutrition caused by NAC may have a negative effect on postoperative recovery. Even if the tumor stage can be reduced, the increased risk of POCs may offset the survival benefit [23]. They often are unable to tolerate and heal the trauma caused by the surgery, let alone the extensive general weakness brought upon them by NAC. As such, adequate preoperative evaluation, treatment of related diseases, and appropriate postoperative recovery strategies must be fully completed before operating on older patients. A retrospective study in China identified BMI ≥ 25 kg/m^2^ as an independent risk factor for POCs [24]. For obese patients, operation difficulties caused by narrow operative space and unclear exposure of important vessels may lead to an increased incidence of complications. Moreover, obese patients may have poorer cardiovascular strength, high blood glucose levels, and poor respiratory function, which are likely to lead to coronary heart disease, poor incision healing, and respiratory failure.

Our study demonstrated that multivisceral resection and long operation time can affect the occurrence of complications. In advanced GC, complete R0 resection is an important factor that improves the prognosis of patients. Therefore, multivisceral resection may be required for patients with invasive tumors. In a phase II trial, multivisceral resection was found to be the only risk factor for complications after NAC (resection vs. no resection, OR = 2.88, 95% CI = 1.13–7.38) [25]. Dias also reported that increased morbidity and lower survival are expected for GC patients undergoing multivisceral resection [26]. OS and disease-free survival (DFS) were lower in these patients (71.5% vs. 55.4%; *p* < 0.001 and 77.8% and 51%; *p* < 0.001; respectively). Meanwhile, narrow operation space in high-BMI patients and more complex surgeries with organ resection would require longer operation time and may result in poorer outcomes. The association of operative time with surgical site infection has been reported in several studies [27,28,29]. In postoperative pathology, it is often found that only a portion of patients undergoing multivisceral resection are confirmed to have tumor invasion, possibly due to the fibroplastic response of the adjacent tumor [30]. Accurate preoperative staging and careful intraoperative operation can avoid unnecessary organ resection, thus reducing the incidences of POCs and improving the prognosis of patients.

The complications were classified according to different criteria in the present study. The Clavien–Dindo classification system improves consistency of reporting results. It is convenient to compare the complications in different periods, different institutions, or different treatments. It is also helpful to reflect the severity of complications more objectively. In our study, we identified multivisceral resection as a factor that may cause more serious complications. Saunders reported that patients with grade III-IV complications had reduced median OS (19.7 vs. 42.7 months; *p* < 0.001) and DFS (18.4 vs. 36.4 months; *p* < 0.001) in a non-leak-related complications group [31]. Additional organs’ resection during gastrectomy can result in increased surgical trauma and impaired related functions of the body, thus affecting postoperative recovery. In advanced GC, tumors sometimes invade adjacent organs, including the pancreas, liver, gallbladder, colon or spleen, and can cause pancreatic fistulas, biliary fistulas, anastomotic leakage or bleeding, which may lead to grade III-IV serious complications. The effect of BMI on the complications of gastric surgery has been discussed above. A high BMI makes surgery difficult, due to a narrower surgical space, which may lead to inflammation-related complications, such as pelvic abscesses. Additionally, patients with high BMI might also have poor respiratory function, resulting in atelectasis, pneumonia, and other complications [32]. In a prospective Chinese cohort study, abdominal obesity was associated with a higher long-term risk of sepsis-related mortality [33]. BMI affect patients’ preoperative health, accurate intraoperative operation, and postoperative recovery. Of note, we found that low preoperative albumin was a risk factor for leakage complications. Hypoalbuminemia was associated with poor tissue healing and decreased tensile strength, because of reduction of collagen synthesis at the surgical site [34]. Even if albumin can be supplemented intravenously or orally after surgery, patients with preoperative hypoalbuminemia are suffering a high risk of anastomotic leakage. This finding may indicate that it is important to improve the patient’s nutritional status before surgery.

Although neoadjuvant therapy has been included in the guidelines for standard treatment of advanced GC, we should not ignore the possible side effects of neoadjuvant therapy. The harms of NAC may even outweigh the benefits in certain groups of patients, so we need to explore the risk factors and intervene in a timely way. If the risk factors for complications are identified, it is possible to consider the balance of risks and benefits to determine appropriate NAC indications. As mentioned in our study, BMI, age, multivisceral resection, operation time, and preoperative albumin level are all factors influencing the occurrence of POCs. In clinical practice, we should actively improve the preoperative nutritional status of patients, so that patients can better tolerate the impacts of surgery. For high-risk patients, the operation time should be shortened as much as possible, and accurate dissection should be performed to avoid unnecessary organ resection. After surgery, patients prone to postoperative complications can also be paid more care, such as closely monitoring the color and amount of fluid from the surgical drain, and abdominal physical examination of patients, so as to detect potential complications in time. Prompt treatment will improve the prognosis of patients.

There are several limitations to this study. First, our study was a retrospective study and conducted at a single center. The number of cases included in the study was small and lacked detailed information. A prospective, large-case, multicenter study is needed to verify the reliability of the results obtained. Second, since the latest patient we followed up underwent operation in 2018, the prognosis of the patient was only calculable up to a 3-year OS, without DFS or other data. We expect to get more prognostic data as the patient population and duration of follow-up increases. The influence of postoperative complications on the recurrence and metastasis of GC may be further explored. Third, the study did not explore the effect of different operative approaches (laparoscopic versus open) or different chemotherapy regimens (SOX versus XELOX) on postoperative complications, which may be useful for clinical decisions. The different operative approaches will affect the time and difficulty of operation. Similarly, the different neoadjuvant chemotherapy regimens will affect the preoperative physical condition of patients and their ability to tolerate surgery. Finally, we do not have genetic information such as MSI in our database. This biological and molecular feature represents a mechanism of resistance to neoadjuvant treatment and has a potential prognostic value that should not be ignored as we move forward in future studies.

## 5. Conclusions

In summary, our study revealed that among advanced GC patients receiving NAC, patients with POCs had a poor prognosis. We also identified the risk factors for complications so that postoperative complications can be avoided in time. Further study is required to provide stronger evidence to inform clinical and policy decisions.

## Figures and Tables

**Table 1:** General characteristics of 176 NAC gastric cancer patients.

Characteristics	No. of Patients	Complication (%)
Gender		
Male	112	22 (19.6)
Female	64	8 (12.5)
Age (years)		
<65	133	15 (11.3)
≥65	43	15 (34.9)
Body mass index (kg/m^2^)		
<25	153	21 (13.7)
≥25	23	9 (39.1)
Extent of gastrectomy		
Subtotal	53	2 (3.8)
Total	123	28 (22.8)
ypStage		
0–II	71	7 (9.9)
III–IV	105	23 (21.9)
ypT category		
T0–T2	40	3 (7.5)
T3–T4	136	27 (19.9)
ypN category		
N0–N1	93	14 (15.1)
N2–N3	83	16 (19.3)
Multivisceral resection		
Yes	47	16 (34.0)
No	129	14 (10.9)
Operation time (hours)		
<5	74	5 (6.8)
≥5	102	25 (24.5)
Preoperative hemoglobin (g/L)		
<120	102	16 (15.7)
≥120	74	14 (18.9)
Preoperative neutrophil (10^9^/L)		
<1.5	34	8 (23.5)
≥1.5	142	22 (15.5)
Preoperative platelet (10^9^/L)		
<100	38	7 (18.4)
≥100	138	23 (16.7)
Preoperative albumin (g/L)		
<40	57	16 (28.1)
≥40	119	14 (11.8)
Postoperative complications		
Yes	30	-
No	146	-

**Table 2:** Univariate and multivariate analysis of POCs.

Characteristics	Univariate Analysis		Multivariate Analysis	
	OR (95% CI)	*p* Value	OR (95% CI)	*p* Value
Gender		0.229		
Male	1.711(0.713–4.106)			
Female	1.000			
Age (years)		0.001		0.003
<65	1.000		1.000	
≥65	4.214(1.845–9.624)		4.920(1.694–14.293)	
Body mass index (kg/m^2^)		0.004		0.005
<25	1.000		1.000	
≥25	4.041(1.554–10.507)		5.907(1.700–20.529)	
Extent of gastrectomy		0.007		0.063
Subtotal	1.000		1.000	
Total	7.516(1.721–32.830)		4.586(0.923–22.779)	
ypStage		0.042		0.560
0–II	1.000		1.000	
III–IV	2.564(1.035–6.352)		1.434(0.427–4.817)	
ypT category		0.080		0.259
T0–T2	1.000		1.000	
T3–T4	3.055(0.876–10.660)		2.664(0.485–14.620)	
ypN category		0.458		
N0–N1	1.000			
N2–N3	1.348(0.613–2.962)			
Multivisceral resection		0.001		0.013
Yes	4.240(1.868–9.623)		3.703(1.320–10.393)	
No	1.000		1.000	
Operation time (hours)		0.004		0.018
<5	1.000		1.000	
≥5	4.481(1.626–12.347)		4.583(1.298–16.186)	
Preoperative hemoglobin (g/L)		0.547		
<120	0.797(0.362–1.756)			
≥120	1.000			
Preoperative neutrophil (10^9^/L)		0.267		
<1.5	1.678(0.673–4.184)			
≥1.5	1.000			
Preoperative platelet (10^9^/L)		0.799		
<100	1.129(0.444–2.874)			
≥100	1.000			
Preoperative albumin (g/L)		0.009		0.086
<40	2.927(1.311–6.533)		2.501(0.878–7.128)	
≥40	1.000		1.000	

**Table 3:** Clavien–Dindo classification in complications group.

Characteristics	Number of Cases	Grade I–II	Grade III–IV	*p* Value
Gender				1.000
Male	22	10	12	
Female	8	4	4	
Age (years)				0.715
<65	15	8	7	
≥65	15	6	9	
Body mass index (kg/m^2^)				0.440
<25	21	11	10	
≥25	9	3	6	
Extent of gastrectomy				1.000
Subtotal	2	1	1	
Total	28	13	15	
ypStage				1.000
0–II	7	3	4	
III–IV	23	11	12	
ypT category				0.586
T0–T2	3	2	1	
T3–T4	27	12	15	
ypN category				0.299
N0–N1	14	5	9	
N2–N3	16	9	7	
Multivisceral resection				0.026
Yes	16	4	12	
No	14	10	4	
Operation time (hours)				1.000
<5	5	2	3	
≥5	25	12	13	
Preoperative hemoglobin (g/L)				0.464
<120	16	6	10	
≥120	14	8	6	
Preoperative neutrophil (10^9^/L)				1.000
<1.5	8	4	4	
≥1.5	22	10	12	
Preoperative platelet (10^9^/L)				1.000
<100	7	3	4	
≥100	23	11	12	
Preoperative albumin (g/L)				0.464
<40	16	6	10	
≥40	14	8	6	

**Table 4:** Inflammation complications group.

Characteristics	Number of Cases	Non-Inflammation	Inflammation	*p* Value
Gender				1.000
Male	22	10	12	
Female	8	4	4	
Age (years)				0.715
<65	15	8	7	
≥65	15	6	9	
Body mass index (kg/m^2^)				0.017
<25	21	13	8	
≥25	9	1	8	
Extent of gastrectomy				1.000
Subtotal	2	1	1	
Total	28	13	15	
ypStage				0.675
0–II	7	4	3	
III–IV	23	10	13	
ypT category				1.000
T0–T2	3	1	2	
T3–T4	27	13	14	
ypN category				0.730
N0–N1	14	6	8	
N2–N3	16	8	8	
Multivisceral resection				1.000
Yes	16	7	9	
No	14	7	7	
Operation time (hours)				1.000
<5	5	2	3	
≥5	25	12	13	
Preoperative hemoglobin (g/L)				0.299
<120	16	9	7	
≥120	14	5	9	
Preoperative neutrophil (10^9^/L)				0.417
<1.5	8	5	3	
≥1.5	22	9	13	
Preoperative platelet (10^9^/L)				0.675
<100	7	4	3	
≥100	23	10	13	
Preoperative albumin (g/L)				0.464
<40	16	6	10	
≥40	14	8	6	

**Table 5:** Leakage complications group.

Characteristics	Number of Cases	Non-Leakage	Leakage	*p* Value
Gender				0.698
Male	22	13	9	
Female	8	4	4	
Age (years)				1.000
<65	15	9	6	
≥65	15	8	7	
Body mass index (kg/m^2^)				0.443
<25	21	13	8	
≥25	9	4	5	
Extent of gastrectomy				1.000
Subtotal	2	1	1	
Total	28	16	12	
ypStage				0.190
0–II	7	2	5	
III–IV	23	15	8	
ypT category				0.070
T0–T2	3	0	3	
T3–T4	27	17	10	
ypN category				0.713
N0–N1	14	7	7	
N2–N3	16	10	6	
Multivisceral resection				0.484
Yes	16	8	8	
No	14	9	5	
Operation time (hours)				0.628
<5	5	2	3	
≥5	25	15	10	
Preoperative hemoglobin (g/L)				0.484
<120	16	8	8	
≥120	14	9	5	
Preoperative neutrophil (10^9^/L)				0.407
<1.5	8	6	2	
≥1.5	22	11	11	
Preoperative platelet (10^9^/L)				0.675
<100	7	4	3	
≥100	23	13	10	
Preoperative albumin (g/L)				0.464
<40	16	6	10	
≥40	14	11	3	

## Data Availability

The data presented in this study are available on request from the corresponding author.
